# Nursing care in anti-N-methyl-d-aspartate receptor encephalitis: A case series

**DOI:** 10.1097/MD.0000000000017856

**Published:** 2019-11-15

**Authors:** Li Yang, Qian Jiang, Hongzhi Guan, Haixin Bo

**Affiliations:** a Department of Neurology; b Department of Nursing, Peking Union Medical College Hospital, Beijing, China.

**Keywords:** anti-*N*-methyl-d-aspartate receptor encephalitis, immunotherapy, nursing care, ovarian teratoma

## Abstract

Anti-*N*-methyl-d-aspartate receptor (anti-NMDAR) encephalitis is a paraneoplastic limbic encephalitis, recently identified.

To summarize our experience in the nursing care of patients with anti-NMDAR encephalitis managed with surgery and pharmacotherapy.

This study included 45 patients treated between July 2015 and November 2016. Laparoscopic oophorocystectomy was performed in 11 female patients with teratomas. Eleven patients required tracheal intubation or tracheotomy and ventilation.

The patients were hospitalized for an average of 25.2 days. The mental and neurological symptoms were significantly relieved 23.6 ± 4.8 days after surgery or immunotherapy. Near-normal function was restored in 11 patients, while 34 patients had varying degrees of dysfunction at discharge. After follow-up of 1 to 18 months, 24 patients were found to have permanent impairments.

Appropriate symptomatic nursing care is required to ensure the safety of patients with anti-NMDAR encephalitis.

## 1 Introduction

Anti-*N*-methyl-d-aspartate receptor (anti-NMDAR) encephalitis is an acute autoimmune condition characterized by abnormal behavior, speech impairment, seizures, movement disorder, decreased consciousness, and autonomic dysfunction.^[1]^ Anti-NMDAR encephalitis is caused by antibodies against NMDA receptors on the surface of hippocampal neurons,^[2]^ and often occurs as a paraneoplastic syndrome associated with ovarian teratoma.^[3]^ It affects males and females of all ages.^[4]^ The condition is potentially fatal.^[5]^ In the United States, the incidence of anti-NMDAR encephalitis rivals that of viral encephalitis.^[6]^ In England, anti-NMDAR encephalitis accounts for 4% of all encephalitis cases.^[7]^ Anti-NMDAR encephalitis was reported for the first time in China by Xu et al in 2010.^[8]^

Despite symptom severity, paraneoplastic anti-NMDAR encephalitis has a better prognosis than most other paraneoplastic encephalitis conditions. The symptoms can be alleviated by first-line treatments (e.g., glucocorticoids, plasma exchange, and intravenous immunoglobulin [IVIg]) or second-line treatments (e.g., cyclophosphamide and rituximab).^[9]^ The nursing care of these patients is challenging because of the complex clinical manifestations, long disease duration, slow recovery, and high risk of recurrence and death. Furthermore, because of the limited information available on the disease, the management of the psychiatric symptoms in critically ill patients with anti-NMDAR encephalitis is difficult.^[10]^

The aim of this study was to report our experience in the nursing care of 45 patients with anti-NMDAR encephalitis from presentation to end of treatment. This study could improve our clinical understanding of this condition and the quality of nursing care offered to patients with anti-NMDAR encephalitis.

## 2 Materials and methods

### 2.1 Patients

This retrospective study included 45 consecutive patients that underwent treatment for anti-NMDAR encephalitis at our hospital between July 2015 and November 2016. We recorded the treatments and nursing care provided to the patients, and assessed the clinical outcome, prognosis, complications, and permanent impairments. The study was approved by the ethics committee of our hospital. Because of the risk to fertility, informed consent was obtained prior to surgery from all patients or their legal representatives.

### 2.2 Preoperative care

Surgical treatment is the only treatment for ovarian teratoma. Routine preoperative care and examinations were carried out, including electrocardiography, chest radiography, blood tests, urine tests, stool tests, blood type, coagulation function, and pelvic B-mode ultrasound. Skin preparation of the abdominal and perineal regions was performed 1 day before operation. The umbilical skin was cleaned and disinfected or the patient was bathed. The patients were instructed to maintain personal hygiene. For vaginal preparation, a 1:40 iodine solution was used in the morning and evening on the day before the operation. The patients were fasted for 12 h before operation. Polyethylene glycol-electrolyte powder or 25% magnesium sulfate was prescribed for bowel preparation. The patient's history of drug allergy was recorded, and a drug allergy test was carried out before the operation. The surgical procedure and main points on which cooperation was required from the patient were explained to the patients, and their questions were answered to alleviate their doubts and concerns about the operation. The patients were instructed to practice deep breathing and effective coughing exercises, and learn how to relieve themselves on a bed before surgery in order to minimize postoperative micturition and defecation difficulties. The surgery was scheduled to avoid the menstrual period. The patients were offered easily digestible semi-fluid food 2 days before operation, and were given liquid food 1 day before surgery. Gas-producing foods (like milk and beans) were avoided. Vulvar and urethral cleaning were performed twice daily.

### 2.3 Early postoperative care

After returning to the ward, the nurses assessed the patients’ condition in details. The patients were asked to lie flat with the head turned to one side, without pillow, for 6 h. They were given low-flow oxygen. The respiratory tract and trachea were kept clean for tracheal intubation and tracheotomy. Blood pressure, pulse, respiration, and blood oxygen were monitored closely. The patients were monitored for hemorrhage from the operation wound or vagina. Care was taken to prevent undue strain on the abdominal cavity drainage tube, and to maintain its patency. The amount, color, and nature of the drainage fluid were noted.

Subcutaneous emphysema is a specific complication of laparoscopic surgery. Owing to increased intra-abdominal pressure during laparoscopy, gas can diffuse from the stomach to the skin, or directly penetrate into the skin (in the case of pneumoperitoneum). Usually, this condition resolves by itself. Abdominal distension typically lasts for 12 to 24 h after surgery, and is mainly caused by the injection of CO_2_ into the abdomen. The CO_2_ gas was suctioned from the abdominal cavity after operation to minimize postoperative distension.

Early mobilization helps intestinal function recovery after operation. Subcutaneous congestion is mainly observed during 12 to 48 h after surgery, and causes darkening of the skin around the incision. This complication is more obvious in patients with abdominal obesity, and is mostly due to the intraoperative pulling of the abdominal skin, which causes rupture of the subcutaneous capillaries. It resolves spontaneously without any treatment.

### 2.4 Medical treatment

Early immunotherapy is crucial for good prognosis of anti-NMDAR encephalitis. IVIg was administered at 0.4 g/kg body weight/day for 5 days (i.e., one cycle). Each patient received 2 to 4 cycles of IVIg treatment in combination with steroids (usually 1 g methylprednisolone for 5 days followed by 60 mg/d metacortandracin, gradually tapered according to the patients’ condition). The IVIg solution was infused at l mL/min for the first 15 min. If no adverse reaction occurred, the infusion rate was gradually increased, not exceeding 3 mL/min. The occurrence of adverse reactions to immunoglobulins is 1%. Glucocorticoid therapy was administered in the form of high-dose methyl prednisone pulse therapy (1, 0.5, and 0.25 g/day for 5 days each) or intravenous dexamethasone (usually 10 or 15 mg/d for 3–5 days, depending on the patient's weight and condition).

Long-term treatment with high doses of hormones causes imbalances in water, electrolytes, carbohydrates, proteins, and lipids. This can result in Cushing syndrome (mainly manifested as centripetal obesity, commonly known as moon face or buffalo hump), acne, hirsutism, atony, hypokalemia, edema, hypertension, and diabetes mellitus.^[11]^ Patients with high-blood pressure or high intraocular pressure were monitored for symptoms such as headache, dizziness, nausea, vomiting, severe eye irritation, and impaired vision. Blood pressure was monitored closely. Attention was paid to osteoporosis symptoms, ulcers, blood sugar changes, symptoms of endocrine disorders, and other side effects.

In addition to the above first-line treatments, second-line treatment with rituximab (Mabthera; Roche Diagnositcs GmbH) was administered if required. When rituximab was administered, the patient's temperature was monitored in order to promptly detect and treat allergic reactions and fever. Thirty minutes before rituximab administration, the patient was given intramuscular injections of 5 mg dexamethasone and 20 mg diphenhydramine along with acetaminophen and bupropion.

Intravenous fluids were administered and the infusion rate was strictly controlled: 50 mg/h for the first 30 min, without exception, and subsequently increased to 50 mg/min. Visits were limited to prevent contagious diseases like colds. Heart rate and breathing were monitored for early detection of cardiac arrest or arrhythmia.

### 2.5 Seizures

Epileptic seizures may occur at any stage during the course of anti-NMDAR encephalitis, but their frequency and intensity decrease with recovery.^[12]^ A spatula was kept readily available at the bedside to prevent tongue biting and respiratory obstruction during seizures. Breathing was kept steady to reduce the risk of aspiration pneumonia due to vomiting and aspiration. The patient's head was tilted to one side, and oxygen was provided in a timely manner. In the event of status epilepticus, care was taken to handle the patient with care and not exert excessive pressure on the patient's body to avoid fractures or joint dislocation. Anti-epileptic drugs were administered as prescribed by the attending physician, including sodium phenobarbital 0.1 to 0.2 g i.m., diazepam 10 mg i.m., or 1 to 2 mL/mg/h midazolam i.v.

### 2.6 Mental and behavioral disorders

In patients with anti-NMDAR encephalitis, abnormal movements can be caused by psychosis, auditory and visual hallucinations, aggression, anxiety, depression, insomnia, catatonia, choreoathetosis, and complex and stereotypic movement disorders.^[13]^ Mental and behavioral disorders are the most common clinical manifestations of anti-NMDAR encephalitis, and are often misdiagnosed as psychosis or schizophrenia. No objects that could be used as a weapon were kept near the patient's bed to prevent self-injury and harm to others. Hot liquids were kept away from the patients to prevent burn wounds. Midazolam and propofol were used for sedation of restless patients. The sedative effect was greater when both drugs were used in combination.

### 2.7 Central hypoventilation

Central hypoventilation has been reported in some cases of brainstem or hypothalamic paraneoplastic syndromes.^[14]^ This condition is characterized by difficult breathing, oxygen desaturation, apnea, decreased partial pressure of arterial blood oxygen, and increased partial pressure of CO_2_. Patients with difficult breathing, oxygen desaturation, or cyanosis of the face, lips, and nails were immediately given oxygen inhalation (mask). Breathing was kept steady, and the patient was laid flat with the head turned to one side to facilitate the discharge of respiratory secretions and vomiting. Artificial airway and mechanical ventilation were used in the event of severe respiratory failure.

### 2.8 Tracheotomy

The tracheal incision dressing was changed once a day. Proper elasticity and length of the catheter were verified. After the tracheal catheter was connected to the ventilator, the catheter was supported against gravity to prevent tracheal compression and necrosis. Long-term compression of the organs or mucous membrane by the tracheal catheter was prevented by discharging air from the catheter for 3 to 5 min every 3 to 4 h. Before air discharge, oropharyngeal suction was applied.

### 2.9 Autonomic dysfunction

In patients with anti-NMDAR encephalitis, autonomic dysfunction can manifest as hyperthermia, urinary and fecal incontinence, hypertension, severe cardiac dysrhythmia, clinically significant cardiac pauses,^[15]^ hypersexuality,^[16]^ and hypersalivation.^[13]^ Untreated autonomic dysfunction can progress to death or permanent disability.^[17]^

For patients with hypersalivation, the mouth and cheeks were cleaned. A disposable plastic bag was opened, placed 2 to 3 cm away from the mouth, and the head was tilted to one side to let the saliva flow in the plastic bag under gravity. The amount of saliva was recorded. The amount of saliva can reach 50 mL/h and 200 to 320 mL/day when the condition is severe.

### 2.10 Patient education

Serum anti-NMDAR antibody and tumor markers were measured regularly. Patients were instructed to take their drugs on time after leaving the hospital. In particular, they were advised to never miss, reduce, or stop taking hormonal drugs, to use potassium and calcium supplements, to take rest, to avoid physical exertion, to undergo rehabilitation training, and to take safety precautions to prevent accidents. For female patients of >12 years of age, a pelvic MRI was carried out every 6 months for 4 years.^[12]^

## 3 Results

### 3.1 General patient information

Of the 45 patients, 12 were male, and 33 were female. They were 11 to 58 years of age. Mean age at onset was 25.1 years. The average hospital stay was 25.2 days (range, 13–56 days).

### 3.2 Clinical symptoms

Precursor symptoms were present in 27 of the 45 (60%) patients, and mainly were symptoms of upper respiratory tract infection such as headache, fever, runny nose, and sore throat.^[18]^ The average interval between precursor symptoms and the initial mental and behavioral symptoms was 12 days. In 28 (62%) patients, mental and behavior disorders were the presenting symptoms.

Of the 45 patients in this study, 34 were agitated, were shouting, and showed anxiety, irritability, tension, insomnia, and auditory and visual hallucinations. During hospitalization, one patient exhibited a jealousy delusion; he firmly believed that his spouse was being unfaithful, and showed aggressive behavior toward her. Nine patients had urinary and fecal dysfunction such as incontinence, dysuria, and constipation. Five patients had tachycardia (maximum heart rate, 180 beats/min). The symptoms are shown in Table 1.

**Table 1:**
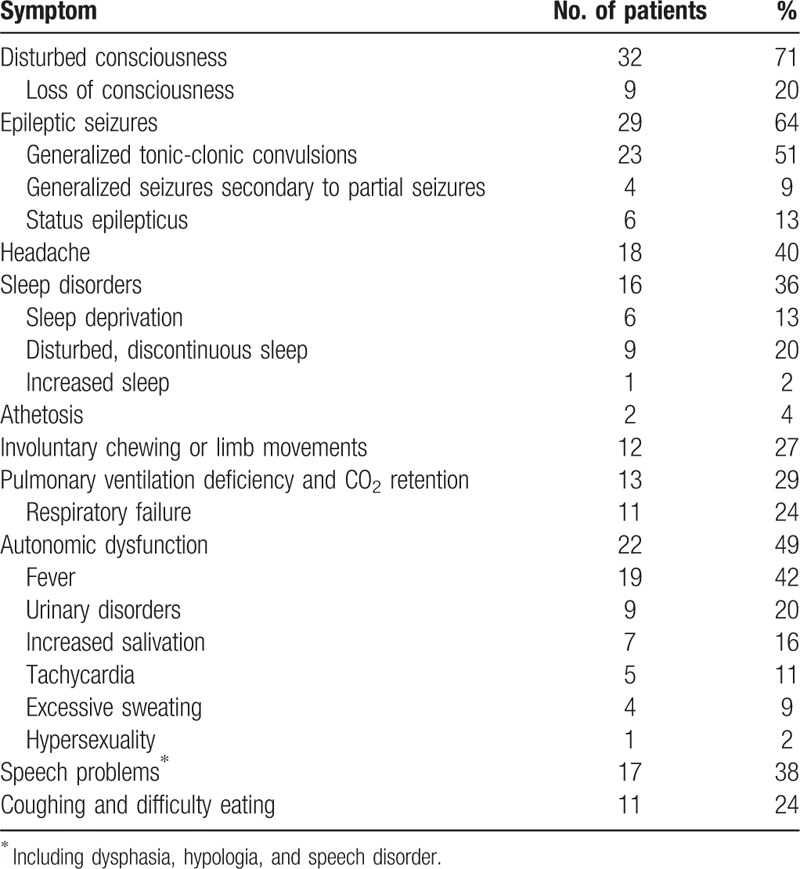
Clinical symptoms in the patient cohort (n = 45).

### 3.3 Investigation findings

Lumbar puncture was performed in all 45 patients. No cerebrospinal fluid (CSF) abnormality was detected in 15 patients (Table 2). The CSF pressure ranged from 90 to 330 mm H_2_O (1 mm H_2_O = 0.0098 kPa). The cytological and biochemical changes in the CSF of patients with anti-NMDAR encephalitis resembled those observed in patients with viral encephalitis. Both CSF pressure and white blood cell count were increased, as was the percentage of multinucleated and mononuclear cells. The protein levels were normal or slightly elevated, while the sugar and chloride levels were mostly normal or slightly low. All 45 patients underwent brain magnetic resonance imaging (MRI) and video EEG after disease onset. The MRI and EEG findings are shown in Table 2.

**Table 2:**
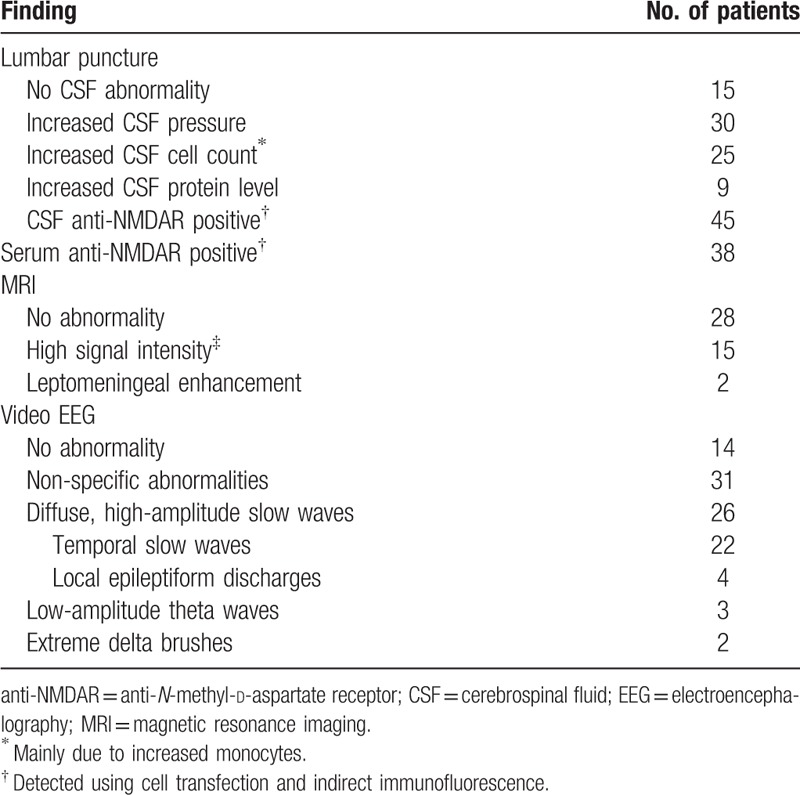
Results of laboratory tests and imaging studies in the patient cohort (n = 45).

### 3.4 Ovarian tumor

All 33 female patients underwent pelvic computed tomography (CT) or transvaginal ultrasound for preoperative tumor screening. Imaging was suggestive of an ovarian teratoma in 11 (24%) patients, all of whom underwent laparoscopic oophorocystectomy. Postoperative pathological examination of the specimen confirmed the presence of ovarian teratoma. One of those patients had bilateral tumors, while the remaining 10 patients had unilateral tumor. In one of these 11 patients, a right ovarian teratoma was found during cesarean section 1 year before disease onset. In another patient, a left ovarian teratoma was resected 6 years before disease onset. Among the 11 patients, seven and four tumors were on the left and right side, respectively. The average tumor diameter was 3.8 cm. Only one patient had an immature grade I teratoma; all other patients had mature teratomas. Mature brain tissue was found in the pathological sections in nine patients.

### 3.5 Treatments and outcome

Drainage tubes were used after operation in five patients and for 1 to 2 days. Dressings were changed for two patients with capillary hemorrhage. All patients received first-line immunotherapy, and five patients received second-line treatment with rituximab after initial therapy because of no improvement. The treatments and outcomes of all 45 patients are shown in Table 3. In 43 patients, the mental and neurological symptoms were relieved after treatment, and 39 of these patients were eventually discharged from the hospital, but 4 were advised to continue in-hospital treatment. The remaining two patients showed no symptomatic improvement, but their families requested discharge from the hospital. There were no deaths. After treatment, normal function was restored in 12 patients, while varying degrees of dysfunction persisted in the remaining patients (e.g., cognitive dysfunction, autonomic nervous dysfunction, motor dysfunction, and/or memory deterioration). All 45 patients were followed by phone for an average of 6 months (range, 1–18 months). Twenty-four patients were left with permanent impairments such as memory impairment, discontinuous agitation, nonresponsiveness, speech impairment, limited physical activity, limb weakness, involuntary cursing, and occasional aggressive behavior.

**Table 3:**
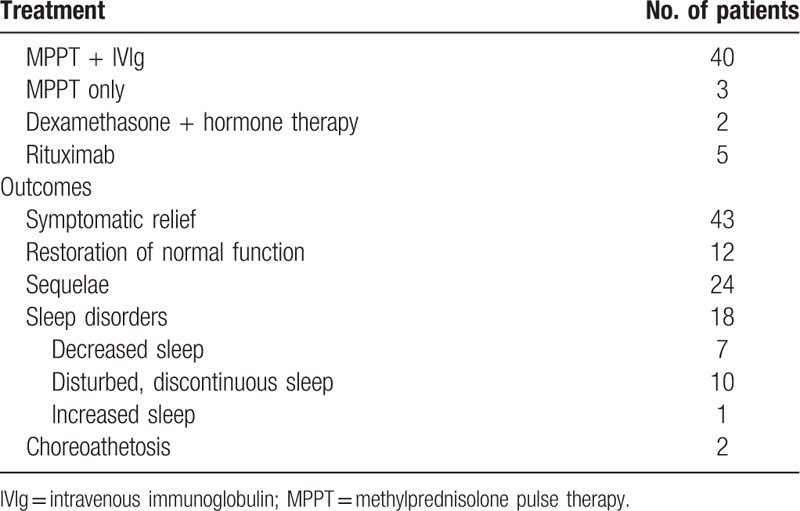
Treatments and outcomes in the patient cohort (n = 45).

Twelve patients who were clearly and continuously restless were given intravenous midazolam or propofol. The 19 patients with fever (highest body temperature, 38.5–39.8°C) were given physical cooling (ice pillow, cold compress on the head, and tepid water sponge baths). These treatments were ineffective in three patients, who were provided with ice blankets. Aspirin-dl-lysine injection was prescribed for 12 patients.

## 4 Discussion

This study aimed to summarize our experience in the nursing care of patients with anti-NMDAR encephalitis. Anti-NMDAR encephalitis is a potentially fatal syndrome that presents with severe psychiatric and neurologic symptoms. Until 5 years ago, limbic encephalitis was considered as a paraneoplastic phenomenon, frequently encountered with lung and testis cancers, and associated with antibodies against neuronal antigens.^[19]^ In a cohort of 577 patients with anti-NMDAR encephalitis, Titulaer et al found that the highest frequency of teratoma-associated disease was among patients of 12 to 45 years of age, with 52% of females >12 years of age having a teratoma.^[20]^ NMDAR antibodies are present in the serum and CSF; occasionally, antibodies are only detectable in the CSF. The main target epitopes are in the NR1/NR2 heteromers of the NMDA receptors.^[21]^ Since the development of the NMDAR assay, some cases of encephalitis of unknown etiology have been reclassified as autoimmune conditions.^[22]^ Over 500 cases of anti-NMDAR encephalitis have been reported in the literature. The California Encephalitis Project (CEP) found that among individuals aged ≤30 years, anti-NMDAR encephalitis is more common than each viral etiologies of encephalitis. Within a 3.5-year period, 32 of 761 (4.2%) cases of encephalitis of uncertain etiology were found to be anti-NMDAR encephalitis.^[6]^ In another study of encephalitis in England, a similar proportion (4.4%) of cases was determined to have been caused by antibodies against NMDA receptors.^[7]^ Although antibodies have been identified in men, approximately 80% of the patients are female. Most tumors are found in females of 12 to 45 years of age. In a recent report, 78% of anti-NMDAR patients in California were white Hispanics and Asian/Pacific Islanders, and 6% were non-Hispanic Caucasians.^[6]^ In addition, tumors associated with anti-NMDAR encephalitis were found more often in Asian and black patients, which suggests a possible genetic risk associated with anti-NMDAR encephalitis.

In most of our patients (62%), the presenting symptoms were mental and behavioral abnormalities, and 40% of our patients were first admitted in a mental hospital and diagnosed with mental disease. Anti-NMDAR encephalitis is often misdiagnosed as mental illness or viral encephalitis.^[4]^ Thus, psychiatrists must be aware of this condition. Involuntary mouth and limb movements (27% patients) were present for 1 to 2 months in our patients. Of the seven patients with obvious hypersalivation, two showed redness of the cheek and neck skin during the initial stages. Salazar et al^[23]^ reported one case of anti-NMDAR encephalitis associated with involuntary movements and hypersalivation; the symptoms were controlled with narcotics and high-dose sedation, and were aggravated after dose reduction. In 2010, Taguchi et al^[24]^ reported a case of anti-NMDAR encephalitis in a 17-year-old patient with ovarian teratoma. The clinical manifestations included abundant salivary secretion for 42 days, described as the “bubbles of a crab,” which was reduced with an anesthetic drug. Salazar et al^[23]^ suggested that hypersalivation may be caused by changes in hypothalamic neurotransmitter levels leading to excessive stimulation of the parasympathetic neurons in the central nervous system. In the present study, 13 patients had pulmonary ventilation deficiency and CO_2_ retention symptoms, including 11 patients with respiratory failure. This is much lower than the frequency of central ventilation failure reported by Dalmau et al^[22]^ (66%). We found that 42% of our patients had fever, which is consistent with the literature (48–86%).^[25]^ Among our patients, 24% had teratomas, of which 91% were benign; the incidence of teratomas in previous reports is 56%.^[22]^

In the present study, 20% of the patients had recurrence, including six patients with one recurrence each, and three patients with two recurrences each. Consistent with this, Dalmau et al^[22]^ reported a recurrence rate of 15% during a mean follow-up of 18 months (range, 1–84 months), including cases of single and multiple recurrences. Only one recurrence occurred among the 11 patients with teratoma in our study, which is consistent with previous reports.^[26]^ Compared with patients with tumors, patients without tumors have higher recurrence rates.^[27]^

Brain MRI in patients with anti-NMDAR encephalitis may be either normal or abnormal.^[28]^ While 38% of our patients had abnormal MRI findings, Dalmau et al^[29]^ reported that 55% of their anti-NMDAR encephalitis patients have MRI abnormalities, which are located in the temporal lobe, hippocampus, corpus callosum, cerebral and cerebellar cortex, frontal lobe, basal ganglia, and brainstem abnormalities. In the present study, 62% of the patients were without MRI abnormalities, and their clinical manifestations included severe mental disorder, epilepsy, involuntary limb movements, and lack of central ventilation, indicating that MRI findings are not always related to clinical severity. Thus, the specific mechanisms underlying anti-NMDAR encephalitis warrant further study.

Considering the long duration, complex clinical manifestations, and life-threatening nature of anti-NMDAR encephalitis, patients with this condition must be treated in an intensive care unit. The disease involves multiple systems and organs, leading to symptoms widely varying among patients, and requiring the intervention of specialists from multiple disciplines. Thus, multidisciplinary communication is crucial to the management of these patients, as this will enable the timely detection and treatment of all types of complications. Psychologists and psychiatrists should be aware of the existence of anti-NMDAR encephalitis in order to avoid misdiagnoses such as viral encephalitis or mental illness. The symptoms of anti-NMDAR encephalitis include hypersalivation, involuntary movements, respiratory impairment, fever, headache, vomiting, mental disorder, and epilepsy. For patients with suspicion of anti-NMDAR encephalitis, lumbar puncture should be performed as early as possible for the detection of anti-NMDA receptor antibody in the CSF. Table 4 summarizes the symptoms and prognosis from multiple case reports and case series.

**Table 4:**
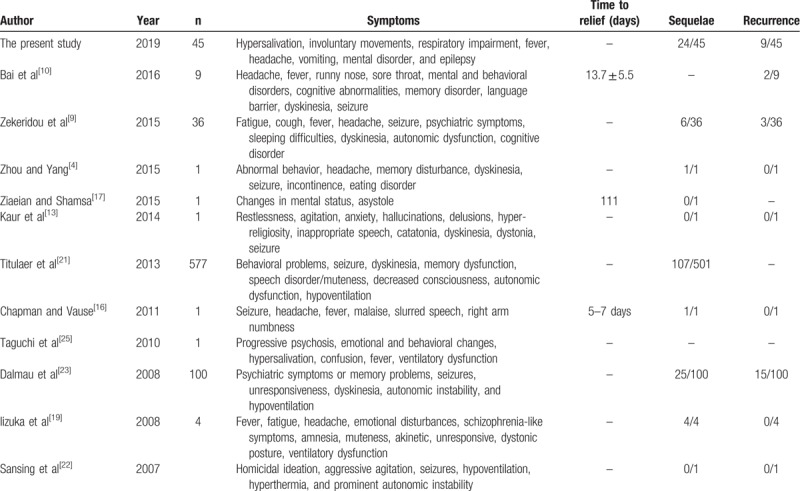
Review of the literature about anti-NMDAR encephalitis.

### 4.1 Limitations

This was a single-center study with a small sample size, and may thus be subject to selection bias. The continuous care of patients after discharge from hospital is the key to the nursing of these patients and a worthy topic for future research.

## 5 Conclusions

Anti-NMDAR encephalitis should be considered among the possible differential diagnoses in patients with an abrupt alteration in mental status. Once a diagnosis is established, patients should be quickly screened for underlying tumors and receive immunosuppressant therapy. Most patients experience sustained remission and near-baseline functioning after rapid, aggressive, and continued immunosuppressant treatment.

## Acknowledgments

None.

## Author contributions

**Conceptualization:** Li Yang, Haixin Bo.

**Data curation:** Li Yang, Qian Jiang, Hongzhi Guan.

**Writing – original draft:** Qian Jiang, Hongzhi Guan, Haixin Bo.
